# The Interdependence between Rainfall and Temperature: Copula Analyses

**DOI:** 10.1100/2012/405675

**Published:** 2012-11-13

**Authors:** Rong-Gang Cong, Mark Brady

**Affiliations:** ^1^Centre for Environmental and Climate Research (CEC), Lund University, Lund S-22362, Sweden; ^2^AgriFood Economics Centre, Department of Economics, Swedish University of Agricultural Sciences, Lund S-22007, Sweden

## Abstract

Rainfall and temperature are important climatic inputs for agricultural production, especially in the context of climate change. However, accurate analysis and simulation of the joint distribution of rainfall and temperature are difficult due to possible interdependence between them. As one possible approach to this problem, five families of copula models are employed to model the interdependence between rainfall and temperature. Scania is a leading agricultural province in Sweden and is affected by a maritime climate. Historical climatic data for Scania is used to demonstrate the modeling process. Heteroscedasticity and autocorrelation of sample data are also considered to eliminate the possibility of observation error. The results indicate that for Scania there are negative correlations between rainfall and temperature for the months from April to July and September. The student copula is found to be most suitable to model the bivariate distribution of rainfall and temperature based on the Akaike information criterion (AIC) and Bayesian information criterion (BIC). Using the student copula, we simulate temperature and rainfall simultaneously. The resulting models can be integrated with research on agricultural production and planning to study the effects of changing climate on crop yields.

## 1. Introduction

 Weather is the key source of uncertainty affecting crop yield especially in the context of climate change [1–3]. For example, Vergara et al. studied the potential impact of catastrophic weather on the crop insurance industry and found that 93% of crop loss was directly related to unfavorable weather [4]. Accurate modeling of multivariate weather distributions would allow farmers to make better decisions for reducing their exposure to weather risk or take advantage of favorable climatic relationships [5]. Among variables relevant to weather, rainfall and temperature are two important factors which have a large effect on crop yield [6–9]. Typically, temperature affects the length of the growing season and rainfall affects plant production (leaf area and the photosynthetic efficiency) [10, 11].

There is a lot of literature studying the effects of temperature and rainfall on crop yield. Erskine and El Ashkar quantified the effect of rainfall on lentil seed yield and found that rainfall accounted for 79.8% of the variance of seed yield [12]. Lobell et al. studied 12 major Californian crops and found rainfall was able to explain more than 60% of the observed variability in yields for most crops [13]. Cooper et al. found that not only the seasonal rainfall totals and their season-to-season variability were important, but also the “within season” variability had a major effect on crop productivity [14], which implies that monthly data is needed in crop production analysis. 

 Muchow et al. found that lower temperature increased the length of time that the maize could intercept radiation and hence grow [15]. Lobell and Asner found a roughly 17% relative decrease in both corn and soybean yield in the USA for each degree of increase in growing season temperature [16]. In summary, it is well established that rainfall and temperature are two important climatic factors affecting agricultural production [17–19]. 

Since temperature and rainfall are critical determinants of crop yield, accurate simulation of temperature and rainfall is important not only for meteorology but also for agricultural economics. However, in reality it is difficult to simulate rainfall and temperature simultaneously due to the interdependence (correlation) between them [20–22]. Spatially, it is generally believed that there exists significant correlation between rainfall and temperature over tropical oceans and land [23]. For example, Aldrian and Dwi Susanto examined the relationship between rainfall and sea surface temperature and found that Indonesian rainfall variability revealed some sensitivity to sea-surface temperature variability in adjacent parts of the Indian and Pacific Oceans [24]. Black also studied the relationship between Indian Ocean sea surface temperature and East Africa rainfall and concluded that strong East African rainfall was associated with warming in the Pacific and Western Indian Oceans and cooling in the Eastern Indian Ocean [25]. 

Temporally, it is generally believed that the correlation between rainfall and temperature changes between months. For example, Rajeevan et al. examined the temporal relationship between land surface temperature and rainfall [26]. They found that temperature and rainfall were positively correlated during January and May but negatively correlated during July. Using annual data Huang et al. also found a negative correlation between rainfall and temperature in Yellow River basin of China [27].

To take the interdependence between rainfall and temperature into account, multivariate probability simulation is needed. Traditionally multivariate probability density functions, however, are generally limited to the multivariate normal distribution or mixtures of it [28]. A possible method that provides an alternative is the copula method. Copulas are advantageous because they can model joint distributions of random variables with greater flexibility both in terms of marginal distributions and the dependence structure [29]. Copulas have been used in financial economics for quite some time [30–32]. However, there are relatively few applications to agricultural weather simulation. 

In respect to temperature and rainfall, AghaKouchak et al. applied two different elliptical copula families, namely, Gaussian and t-copula, to simulate the spatial dependence of rainfall and found that using the t-copula might have significant advantages over the well-known Gaussian copula particularly with respect to extremes [33]. Serinaldi also studied the spatial dependence of rainfall and confirmed that only positive contemporaneous pairs of rainfall observations correctly described the intersite dependence [34]. Laux et al. highlighted the importance of pretreatment of meteorological data in the copula modeling process [35]. Laux et al. used the Clayton copula to construct the bivariate distribution of drought duration and intensity [36]. Similar applications of the Clayton copula can also be found in the studies of Favre et al. and Shiau et al. [37, 38]. Furthermore, they raised the question as to which copula model best fitted the empirical data. The only literature concerning the application of copula simulation to model the interdependence between temperature and rainfall up to now is that of Schölzel and Friederichs [39]. They used a simple statistical model based on the copula approach to describe the phenomenon that cold periods were accompanied by small precipitation amounts. 

Inspired by Dupuis's study on hydrological random variables [40], the purpose of this paper is to illustrate the pretreatment process of meteorological data, demonstrate the application of different copulas to modeling of joint distributions of rainfall and temperature, select the most suitable copula function according to information criteria, and finally simulate rainfall and temperature simultaneously. 

## 2. Materials and Methods

### 2.1. Study Area

 Scania is Sweden's southernmost province and one of Northern Europe's most fertile farming districts with the main crops being winter wheat, rapeseed, sugar beets, and barley. As Scania is surrounded by water on three sides (the Baltic Sea, the Kattegat Sea, and the Öresund Sound), it has a maritime climate, especially along the south and east coasts. The winters are mild (few days of snow), but the summers are similar to those in the rest of southern Sweden.

### 2.2. Data Collection and Preliminary Analysis

Monthly temperature and rainfall data for Scania from 1961 to 2010 was obtained from the Swedish Meteorological and Hydrological Institute. 

#### 2.2.1. Temperature

**Figure 1:**
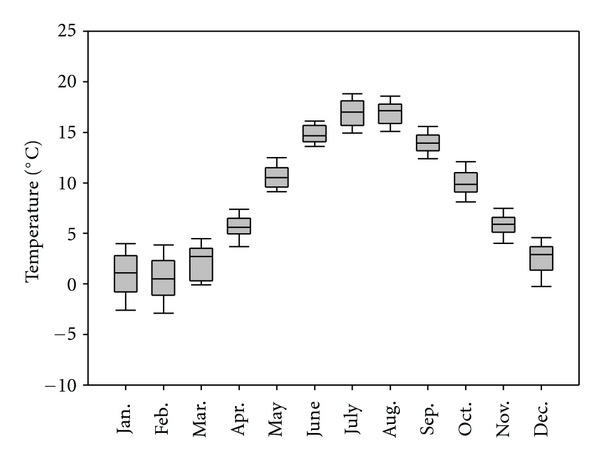
Monthly average temperature in Scania, Sweden, from 1961 to 2010. Note: the boundary of the box closest to zero indicates the 25th percentile, a line within the box marks the median, and the boundary of the box farthest from zero indicates the 75th percentile. Whiskers (error bars) above and below the box indicate the 90th and 10th percentiles.

Monthly average temperature in Scania shows a clear seasonal cycle from 1961 to 2010 (Figure 1). The average temperature usually reaches its peak in July and its bottom in February. From April to November, the average temperature is always above 0°C. The variability of average temperature in January and February is though relatively large. Some descriptive temperature statistics are listed in Table 1.

#### 2.2.2. Rainfall

**Figure 2:**
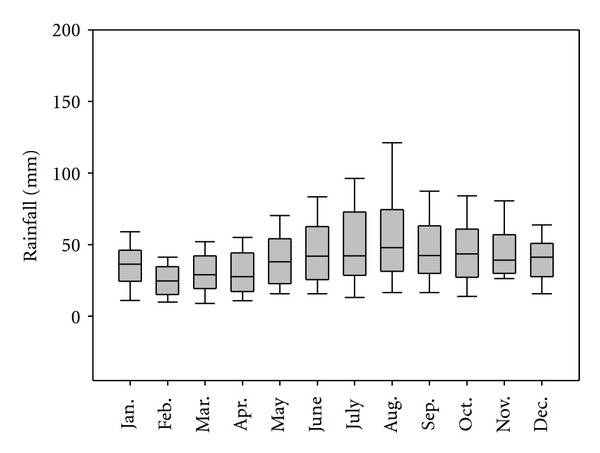
Monthly total rainfall in Scania, Sweden, from 1961 to 2010. Note: the boundary of the box closest to zero indicates the 25th percentile, a line within the box marks the median, and the boundary of the box farthest from zero indicates the 75th percentile. Whiskers (error bars) above and below the box indicate the 90th and 10th percentiles.

Compared with temperature, monthly total rainfall in Scania does not show a clear seasonal cycle from 1961 to 2010. From June to November, the average monthly total rainfall is relatively high (Figure 2). Some descriptive rainfall statistics are listed in Table 2.

#### 2.2.3. The Relationship between Rainfall and Temperature

The physical rationale behind the relationship between rainfall and temperature is that rainfall may affect soil moisture which may in turn affect surface temperature by controlling the partitioning between the sensible and latent heat fluxes [41]. Because the sample data is non-Gaussian distributed and skewed, the Kendall correlation coefficient is employed to calculate the correlation between monthly rainfall and temperature. It is found that there are negative correlations between rainfall and temperature from April to July and in September (at the 10% confidence level) (Table 3).

### 2.3. Methods

 Here we use the copula functions to model the interdependence between the probability distributions of a certain month's temperature and rainfall. Let *X* and *Y* be continuous random variables representing temperature and rainfall, with cumulative distribution functions *F*_*X*_(*x*) = Pr(*X* ≤ *x*) and *G*_*Y*_(*y*) = Pr(*Y* ≤ *y*), respectively. Following Sklar [42], there is a unique function *C* such that

(1) $$\begin{array}{c} \text{Pr}\left(X\leq x,Y\leq y\right)=C\left(F\left(x\right),G\left(y\right)\right), \end{array}$$

where *C*(*u*, *v*) = Pr(*U* ≤ *u*, *V* ≤ *v*) is the distribution of the pair (*U*, *V*) = (*F*(*X*), *G*(*Y*)) whose margins are uniform on [0,1]. The function *C* is called a copula. As argued by Joe [43] and Nelsen [44] among others, *C* characterizes the dependence in the pair (*X*, *Y*). There are many parametric copula families available, which usually have parameters that control the strength of dependence. Among these, five families of commonly used copulas are considered. They are listed in Table 4, along with their parameter ranges. The first three are Archimedean [43] and the last two are metaelliptical [45].

After calculating the parameters of each copula, it is necessary to decide which family is the best representation of the dependence structure between the variables of interest. There are a few techniques to select the best copula. One of them is based on distance measures pertaining to the distributions of the candidate models (copulas) and the empirical distribution of the data [46, 47]. Alternative methods include likelihood ratio tests and approaches related to information criteria [31], such as Akaike [48] and Schwarz's Bayesian [49] Information Criteria. Information criteria are adopted here because they can describe the tradeoff between bias (accuracy) and variance (complexity) in model construction. The Akaike information criterion (AIC) is a measure of the relative goodness of fit of a statistical model. Its definition is

(2) $$\begin{array}{c} \text{AIC}=2k-2\ln\left(L\right), \end{array}$$

where *k* is the number of parameters in the copula and *L* is the maximized value of the likelihood function for the copula. The Bayesian information criterion (BIC) was developed by Schwarz using Bayesian formalism. Its definition is

(3) $$\begin{array}{c} \text{BIC}=-2\ln\left(L\right)+k\ln\left(N\right), \end{array}$$

where *N* is the sample size.

## 3. Results and Discussion

**Figure 3:**
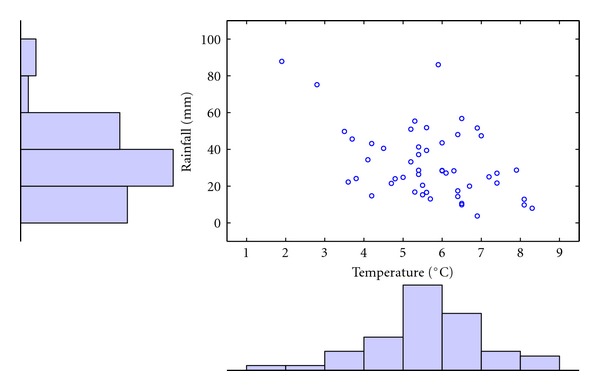
Temperature and rainfall in April from 1961 to 2010.

Temperature and rainfall data in April from 1961 to 2010 is employed as an example to demonstrate the modeling process (Figure 3). There is a significant negative relationship (Kendall correlation coefficient is −0.27, *P*-value = 0.007) between temperature and rainfall in April. Temperature has negative skewness (−0.35) and rainfall has positive skewness (1.07), which may cause a heteroscedasticity problem when fitting the model [50]. Following Kim and Ahn [51], the temperature and rainfall data are log-transformed to remove this effect. The logarithmic transformation for the data is invertible, which will not affect the fitting results.

**Figure 4:**
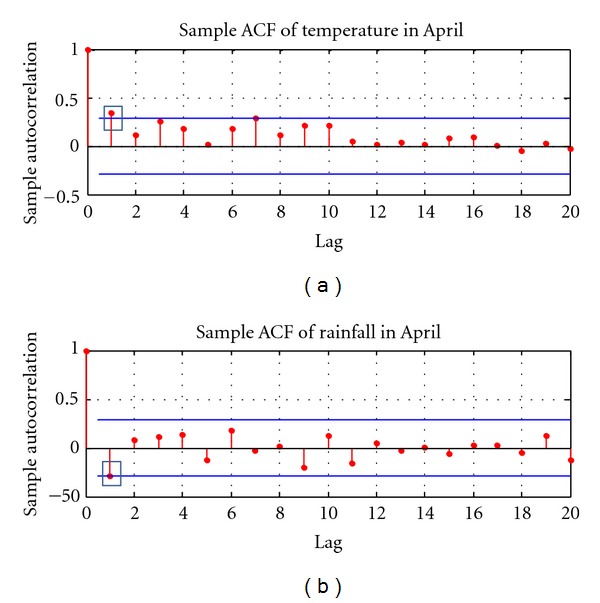
Sample autocorrelation function (ACF) of temperature and rainfall in April from 1961 to 2010.

Following Benth and Šaltyte-Benth's instructions [52], the time series of temperature and rainfall are tested for autocorrelation using the *Q*-statistics (Figure 4). Autocorrelation describes the correlation between values of temperature (or rainfall) at different points in time, as a function of the time difference. The presence of autocorrelation increases the variances of residuals and estimated coefficients, which reduces the model's efficiency. The Ljung-Box *Q* test is a type of statistical test of whether autocorrelations of a time series are different from zero [53]. The *Q*-statistics is defined as follows:

(4) $$\begin{array}{c} Q=N\left(N+2\right)\sum_{a=1}^{h}\frac{{\hat{p}}_{a}^{2}}{N-a}, \end{array}$$

where ${\hat{p}}_{a}^{2}$ is the sample autocorrelation at lag *a*, and *h* is the number of lags being tested. The first-order autocorrelations are found to be strong both for temperature (*Q*-stat = 6.32, *P* value = 0.01) and rainfall (*Q*-stat = 4.52, *P* value = 0.03), as shown in Figure 4. 

Therefore, an AR(1) model is used to eliminate the autocorrelation in the series as follows:

(5) tempet=0.48+0.35×tempet−1+εt        (4.7∗∗)(2.56∗∗),raint=1.85−0.29×raint−1+μt      (9.06∗∗)(−2.1∗∗).

Note that the numbers in the bracket are *t*-values and **stands for the statistical significance at the 95% confidence level. 

**Figure 5:**
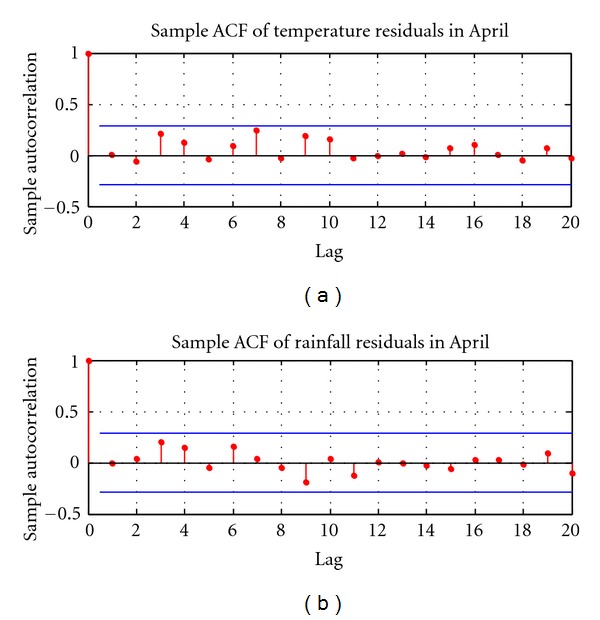
Sample autocorrelation function (ACF) of AR adjusted temperature and rainfall in April.

Residuals *ε*_*t*_ and *μ*_*t*_ are tested where only weak autocorrelations are found (Figure 5). 

In addition to autocorrelation, time trends are also found in the series of *ε*_*t*_ and *μ*_*t*_. Based on Manton et al.'s research [54], the time trends should be removed from the series to obtain a stationary process. The functions used to detrend the time series are

(6) εt=−0.08+0.0032×t+φt   (−2.65∗∗)(3.04∗∗),μt=0.17−0.007×t+γt   (2.3∗∗)(−2.65∗∗).

**Figure 6:**
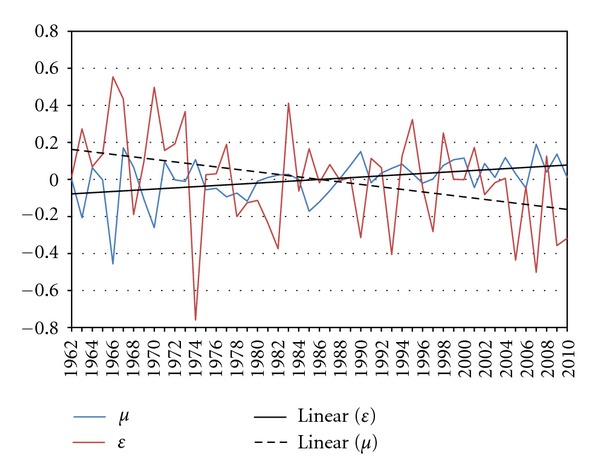
Residuals for AR adjusted temperature and rainfall in April.

**Figure 7:**
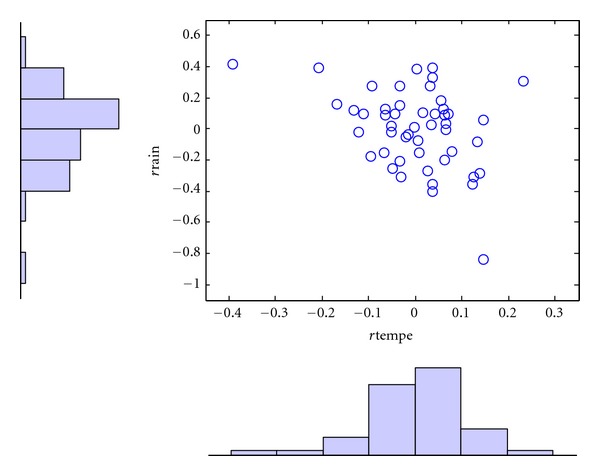
Scatters of residuals for trend adjusted temperature and rainfall in April.

We find that temperature has an increasing trend and rainfall has a decreasing trend in April from 1961 to 2010 (Figure 6). The annual rate of increase in temperature in April is 0.0032°C and decrease in rainfall is 0.007 mm per year. The trend adjusted data are shown in Figure 7 where *r*tempe_*t*_ and *r*rain_*t*_ are used to represent the corrected values of *φ*_*t*_ and *γ*_*t*_, respectively.

The residuals for the trend adjusted variables have negative skewness: temperature (−1) and rainfall (−0.7). Based on the inference for the margins (IFM) [55], the parameter estimates and model evaluation indices for each copula for *r*tempe_*t*_ and *r*rain_*t*_ are presented in Table 5.

The log-likelihood ratio is largest and the AIC and BIC are smallest for the student copula, which means that the student copula is the most suitable model. 

**Figure 8:**
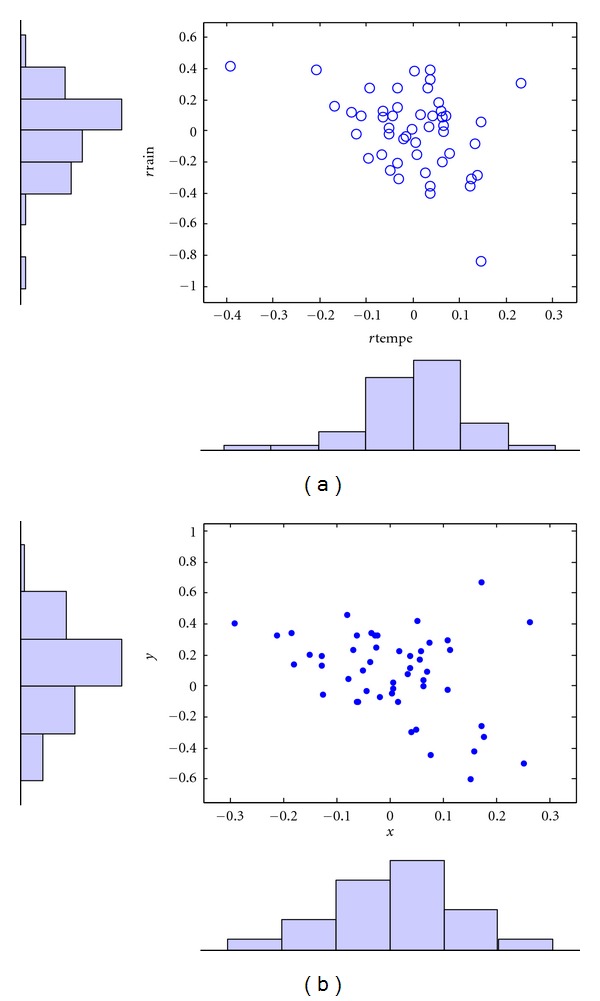
Scatter plots of real residuals (a) and student-based copula simulated residuals (b).

A comparison of the real and simulated residuals of temperature and rainfall is shown in Figure 8.

Since the purpose of this paper is to develop a copula model of the bivariate distribution of rainfall and temperature that can be used in simulation studies, the accuracy of the resulting model is of utmost importance. Although Table 5 has provided some statistical support for the model and Figure 8 has given some visual evidence, the contours of the cumulative distribution functions can best show the difference between the real and simulated data.

**Figure 9:**
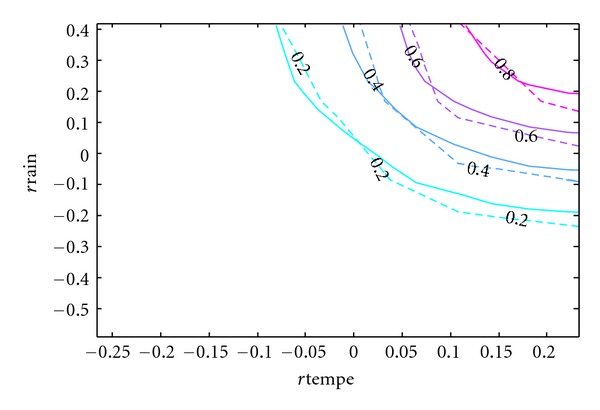
Real versus Gauss fitted CDF. Note: The dashed lines are the real CDFs while the solid lines are the simulated CDFs.

**Figure 10:**
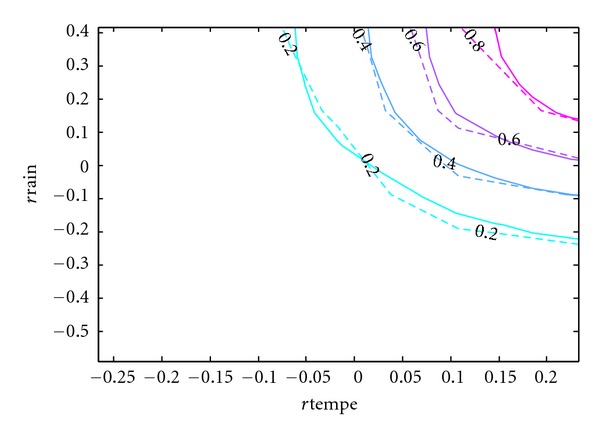
Real versus student fitted CDF. Note: The dashed lines are the real CDFs while the solid lines are the simulated CDFs.

**Figure 11:**
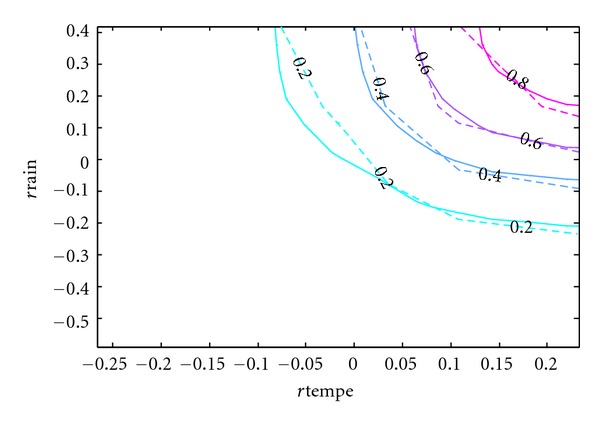
Real versus Clayton fitted CDF. Note: The dashed lines are the real CDFs while the solid lines are the simulated CDFs.

**Figure 12:**
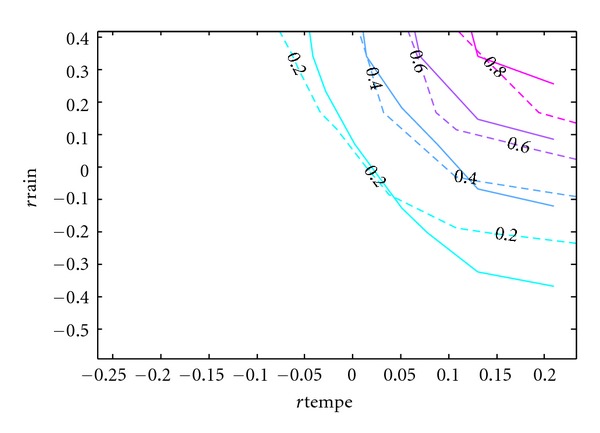
Real versus Frank fitted CDF. Note: The dashed lines are the real CDFs while the solid lines are the simulated CDFs.

**Figure 13:**
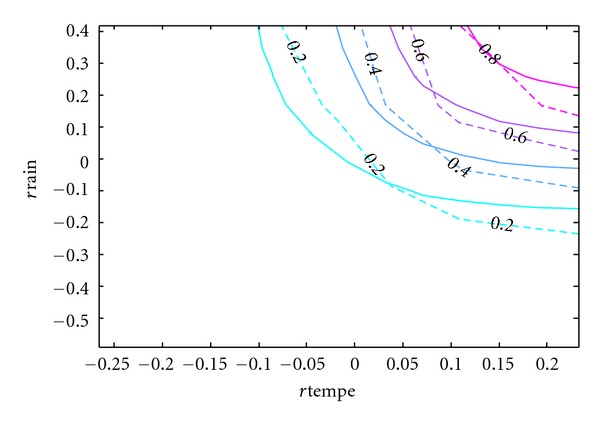
Real versus Gumbel fitted CDF. Note: The dashed lines are the real CDFs while the solid lines are the simulated CDFs.

In Figures 9, 10, 11, 12, and 13, the contours of the cumulative distribution functions (CDFs) for the real and simulated data from the five copula models are plotted to visualize the difference or similarity in the distributions as the case may be. It is found that the student copula model fits the real data best according to the similarity of the contour lines. Consequently the student copula is the best choice of model according to all our criteria.

**Figure 14:**
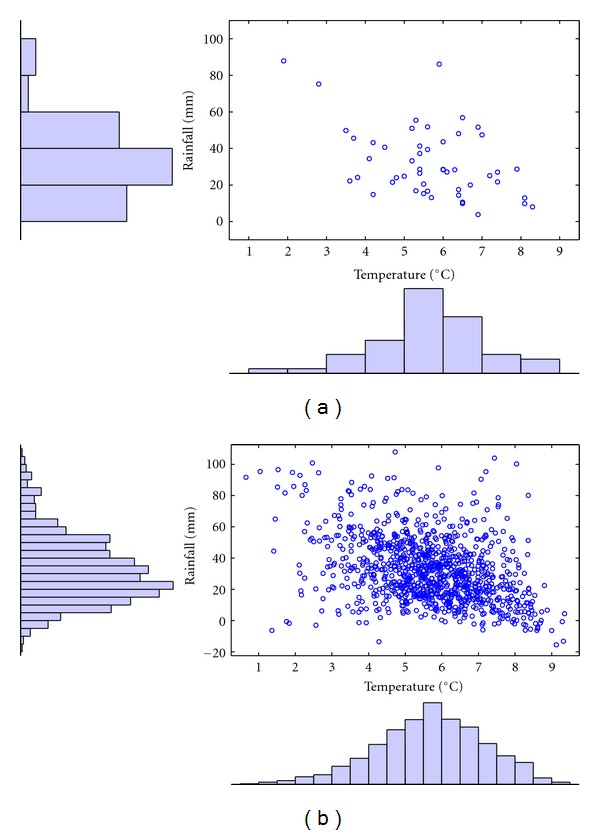
Real (a) and student-based copula simulated (b) temperature and rainfall data for Scania in April.

Based on the estimated parameters, 1,000 draws are made from the Student copula model. The simulated data is then transformed to the original scale and compared with the real data in Figure 14.

## 4. Conclusions

 This paper presents a copula-based methodology for modeling the joint distribution of temperature and rainfall, which are of utmost importance for agricultural production especially in the context of climate change. Copulas have been used extensively in the financial literature, but have not been widely used in weather simulation. The copula approach provides a powerful and flexible method to model multivariate distributions and thus goes beyond joint normality, regression, and mean-variance criterion. Accurate simulation of weather events may help to improve risk management in agricultural planning.

 A shortcoming of the copula method is the arbitrariness of the selection of a particular copula. The main purpose of this paper is to present a complete copula modeling framework to model the interdependence of rainfall and temperature. In contrast to Schölzel and Friederichs [39], we compare different copulas and show how to select the optimal copula based on information criteria (AIC and BIC). The advantage of this approach is that it does not require any assumptions and is primarily data driven thus minimizing the subjectivity introduced by the researcher. The model selection criteria indicate that the Student copula produces the best model to simulate the dependence structure between rainfall and temperature in Scania, Sweden.

 Although the month of April was chosen as our working example, we have also tested the data for other months with similar results. The study is only based on meteorological data for a single region. The most suitable copula family for rainfall and temperature might change from one region to another due to differences in geographical and geophysical conditions. Our approach however can be applied in studies of other parts of the world to select the most appropriate copula model. A potentially valuable extension of this research is to connect the analysis with crop production planning and agricultural economics. If the relationship among temperature, rainfall, and crop yield can be determined, then it could be used in developing risk reducing strategies for farmers, something which will become increasingly important in the face of climate change. This is the focus of our ongoing research.

## Figures and Tables

**Table 1:** Descriptive statistics for monthly average temperature from 1961 to 2010 (unit: °C).

	Jan.	Feb.	Mar.	Apr.	May	June
Maximum	5.10	5.20	5.80	8.30	13.10	18.00
Minimum	−5.20	−5.20	−2.30	1.90	8.40	12.00
Mean	0.83	0.54	2.21	5.68	10.58	14.78
Standard deviation	2.48	2.39	1.85	1.36	1.20	1.07
Variation coefficient	2.99	4.44	0.84	0.24	0.11	0.07

	July	Aug.	Sep.	Oct.	Nov.	Dec.

Maximum	21.00	21.50	16.90	13.20	8.30	7.10
Minimum	13.90	14.60	11.60	7.60	2.70	−2.80
Mean	16.99	17.04	13.98	10.03	5.80	2.49
Standard deviation	1.59	1.45	1.20	1.33	1.27	1.88
Variation coefficient	0.09	0.09	0.09	0.13	0.22	0.76

**Table 2:** Descriptive statistics for monthly total rainfall from 1961 to 2010 (unit: mm).

	Jan.	Feb.	Mar.	Apr.	May	June
Maximum	70.00	50.00	73.50	87.90	90.60	123.3
Minimum	1.00	5.00	3.30	3.80	6.30	0.1
Mean	35.19	25.14	30.07	32.20	39.74	46.28
Standard deviation	17.07	11.38	16.25	18.86	20.46	26.82
Variation coefficient	0.49	0.45	0.54	0.59	0.51	0.58

	July	Aug.	Sep.	Oct.	Nov.	Dec.

Maximum	147.60	189.90	161.90	106.30	95.00	106.00
Minimum	7.40	5.70	7.30	4.50	17.00	4.80
Mean	51.15	58.33	49.20	45.90	45.73	40.80
Standard deviation	30.76	39.97	31.48	24.26	19.65	19.03
Variation coefficient	0.60	0.69	0.64	0.53	0.43	0.47

**Table 3:** Correlation analysis for monthly temperature and rainfall from 1961 to 2010.

	Jan.	Feb.	Mar.	Apr.	May	June
Kendall correlation coefficients	0.12	0.13	0.07	−**0.27**	−**0.3**	−**0.17**
*P* value	0.22	0.19	0.49	**0.007**	**0.002**	**0.08**

	July	Aug.	Sep.	Oct.	Nov.	Dec.

Kendall correlation coefficients	−**0.3**	−0.02	−**0.19**	−0.13	−0.02	0.09
*P* value	**0.002**	0.84	**0.06**	0.19	0.85	0.37

**Table 4:** Five families of copulas.

Family	*C*(*u*, *v*)	Range of *θ*
Normal	*N*_*θ*_ (Φ^−1^(*u*), Φ^−1^(*v*))	[−1, 1]
Student	*T*_*θ*, *γ*_ (*T*_*γ*_^−1^(*u*), *T*_*γ*_^−1^(*v*))	[−1, 1]
Clayton	(*u* ^−*θ*^ + v^−*θ*^ − 1)^−1/*θ*^	(0, ∞)
Frank	−*θ* ^−1^ln{1 + (*e* ^−*θu*^ − 1)(*e* ^−*θv*^ − 1)/(*e* ^−*θ*^ − 1)}	(−∞, ∞)
Gumbel	exp{−[(−ln⁡*u*)^*θ*^ + (−ln⁡*v*)^*θ*^]^1/*θ*^}	[1, ∞)

Φ: cumulative distribution function (CDF) of a *N*(0,1).

*N*_*θ*_: CDF of a standard bivariate normal distribution with Pearson

correlation *θ*.

*T*_*γ*_: CDF of a student distribution with *γ* degrees of freedom.

*T*_*θ*,*γ*_: CDF of a bivariate student distribution with *γ* degrees of freedom.

Source: [46].

**Table 5:** Results of different copula models for temperature and rainfall in April.

	Normal	Student	Clayton	Frank	Gumbel
*θ*	−0.34	−0.31	0.001	0.001	1.1
Log likelihood	3.05	4.11	−0.0007	−0.0002	−1.86
AIC	−6.06	−8.15	0.042	0.041	3.75
BIC	−6.02	−8.07	0.081	0.08	3.79
